# Adherence to Antimicrobial Inhalational Anthrax Prophylaxis among Postal Workers, Washington, D.C., 2001

**DOI:** 10.3201/eid0810.020331

**Published:** 2002-10

**Authors:** Mariaelena D. Jefferds, Kayla Laserson, Alicia M. Fry, Sharon Roy, James Hayslett, Laurence Grummer-Strawn, Laura Kettel-Khan, Anne Schuchat, selected members of the Centers for Disease Control and Prevention Anthrax Adherence Team

**Affiliations:** * Centers for Disease Control and Prevention, Atlanta, Georgia, USA

**Keywords:** adherence, *Bacillus anthracis*, bioterrorism, antimicrobial prophylaxis, compliance

## Abstract

In October 2001, two envelopes containing *Bacillus anthracis* spores were processed at the Washington, D.C., Processing and Distribution Center of the U.S. Postal Service; inhalational anthrax developed in four workers at this facility. More than 2,000 workers were advised to complete 60 days of postexposure prophylaxis to prevent inhalational anthrax. Interventions to promote adherence were carried out to support workers, and qualitative information was collected to evaluate our interventions. A quantitative survey was administered to a convenience sample of workers to assess factors influencing adherence. No anthrax infections developed in any workers involved in the interventions or interviews. Of 245 workers, 98 (40%) reported full adherence to prophylaxis, and 45 (18%) had completely discontinued it. Experiencing adverse effects to prophylaxis, anxiety, and being <45 years old were risk factors for discontinuing prophylaxis. Interventions, especially frequent visits by public health staff, proved effective in supporting adherence**.**

In October 2001, two letters with *Bacillus anthracis* spores were mailed to offices on Capitol Hill, Washington, D.C. Both letters were processed at the Washington, D.C., Processing and Distribution Center (DCPDC) of the U.S. Postal Service (USPS). Inhalational anthrax developed in four DCPDC postal workers; two died. More than 2,000 workers and business visitors to the private work areas of DCPDC were potentially exposed to aerosolized *B. anthracis* spores during October 12–21 (1,2). To prevent inhalational anthrax, 60 days of antimicrobial therapy was recommended (primary: ciprofloxacin 500 mg/orally twice a day or doxycycline 100 mg/orally twice a day; alternative: amoxicillin 500 mg/orally twice a day).

Although inhalational anthrax most often develops in the first 7–10 days after exposure, incubation periods as long as 43 days have been reported in Sverdlovsk, Russia (3); in animal studies, inhalational anthrax occurred after 58 days despite 30 days of antimicrobial therapy (4). Therefore, completion of the full 60 days of prophylactic antimicrobial therapy was essential for all postal workers potentially exposed to *B. anthracis* spores at the DCPDC.

Adherence to long-term drug regimens is problematic, and multiple factors influence adherence status, such as regimen factors (e.g., number of pills needed daily), structural factors (e.g., ability to access drugs), individual factors (e.g., cognitive limitations, depression), and health-care provider factors (e.g., ability to listen to and communicate effectively with patients) (5–10). Among the DCPDC workers, typical adherence issues associated with short-course antimicrobial therapy were complicated by the high levels of stress associated with the bioterrorism event and the illnesses and deaths of coworkers, stigma from other postal workers and community members because of erroneous concerns that DCPDC workers were contagious, and the relatively longer duration and potential adverse effects associated with the therapy. The DCPDC facility was closed October 21, 2001, and employees were displaced to work in other area mail facilities, contributing to ongoing disruptions of the workers’ daily lives and further complicating adherence. Last, the dynamic nature of the bioterrorist event created a system of evolving health-risk communication that, combined with the many inconsistent sources of information about the event and anthrax, contributed to confusion and misinformation.

 In response to the first bioterrorism-related outbreak of inhalational anthrax in the United States, strategies to promote adherence to antimicrobial prophylaxis among more than 2,000 DCPDC workers were rapidly implemented. To facilitate future adherence activities in similar events, we evaluated the interventions that were used to support adherence and examined the factors that influenced adherence to the prophylactic regimen in DCPDC workers.

## Methods

### Qualitative Data Collection

Qualitative data were collected from open-ended interviews (i.e., ones in which interviewer writes down exact responses of interviewee) with convenience samples of the postal worker population throughout the 60-day period to develop and evaluate the interventions and to collect information on the determinants of adherence. The findings from the qualitative interviews were used to develop and validate the close-ended questions (i.e., those with a defined set of answers to choose from, such as yes or no) included in the quantitative survey questionnaire. Information was collected through observation, one-on-one contact, informal small group discussions, and focus group interviews with workers, as well as through interactions with USPS management, worker union representatives, and USPS Employee Assistance Program personnel.

Two staff members from the Centers for Disease Control and Prevention (CDC) conducted five focus group interviews with DCPDC workers during December 13–16, 2001. DCPDC shift supervisors selected six to eight workers to participate in each focus group. During the interviews, workers’ responses were noted verbatim on a large flip chart visible to participants at all times. The first author also carried out individual qualitative open-ended interviews during routine interactions with workers throughout December 2001.

The first author conducted all analyses. Notes were immediately reviewed for accuracy at the completion of all interviews and entered into a word-processing software program. Qualitative analysis included several rounds of coding by subject or theme, as well as content analysis and comparison of responses across groups. Analysis focused on both commonly repeated themes (reported by at least 50% of the respondents) and rare points of view.

### Interventions to Promote Adherence

To develop appropriate adherence interventions, we obtained support from the USPS management, Employee Assistance Program, and postal service unions. We conducted open-ended interviews with postal workers from various jobs and shifts and incorporated known adherence strategies (5,6,8,10,11) to develop interventions.

Public health staff carried out repeated group question-and-answer sessions and informal contact with workers. These sessions consisted of large and small group and one-on-one interactions to counsel workers. Motivational messages were distributed through the USPS communication infrastructure. In addition, several types of written materials were distributed at the worksite and to workers’ homes, including booklets of frequently asked questions about anthrax and antimicrobial therapy, antimicrobial pocket guides with calendar memory aids, and handouts describing ways to minimize stress and recognize the known adverse effects of antimicrobial therapy, such as gastrointestinal upset and yeast infection. Posters and table tents, both with motivational messages, were placed in the workplace. We also provided a letter for workers to take to their personal health-care provider clarifying which area postal workers needed extended prophylaxis and the recommended regimens. This letter was also distributed directly to area health-care providers. Further, after free antimicrobial agents were no longer available, access to antimicrobial agents and reimbursements was facilitated. Finally, clinical team members and a local health-care provider answered specific questions about adverse effects or potential drug interactions, and the local health-care provider consulted with workers free of charge.

 In addition, multiple Morbidity and Mortality Weekly Reports (12–14), Health Alert Network alerts, and live broadcasts were disseminated throughout the prophylaxis period to give health-care providers detailed information on which groups needed extended prophylaxis, the recommended regimens, and clinical signs of inhalational anthrax disease.

### Quantitative Survey

At five mail facilities, trained interviewers administered a close-ended questionnaire to a convenience sample of all DCPDC employees working the day shift (7 a.m.–3 p.m.) on December 18–20, 2001, days 57–60 of the 60-day regimen. Prophylaxis was first offered October 21, 2001, and most workers picked up prophylaxis on October 22 or 23, 2001. Most (80%) of the displaced DCPDC employees worked at these five facilities. Compared with the day shift, more employees work the swing shift and night shift, when the mail collected during the day is processed.

The questionnaire collected information on demographic characteristics, adherence behaviors, enablers and obstacles to adherence, and information about the implementation of interventions. To assess adherence, workers were asked to respond to five questions located throughout the survey. (For example, “Are you still taking antibiotics for anthrax?” [Possible responses: No, Yes, Declined] and “If you forgot to take any of your pills yesterday, how many pills did you miss?” [Possible responses: None, One, Two, Three.).

Because we were interested in adherence to the recommendation to complete 60 days of prophylaxis, workers were divided into one of three categories. Adherence was defined as full if workers reported they continuously took their antimicrobial therapy throughout the 60-day period, never reduced their dosage, and did not forget any pills the previous day. Adherence was defined as intermediate if workers reduced the dosage, forgot a pill the previous day, or stopped their antimicrobial therapy and restarted at least once. Adherence was defined as discontinued if workers stopped their antimicrobial therapy and never restarted.

 To analyze predictors of nonadherence, we carried out a three-step logistic regression modeling procedure. First, we modeled overall nonadherence (intermediate adherence and discontinued groups combined) compared with full adherence. For this model, we were interested in understanding the differences between those workers who were fully adherent and those who were not fully adherent, including workers who completely discontinued therapy. Second, we modeled intermediate adherence compared with full adherence. For this model, we were interested in understanding the differences between those who were nonadherent but who had not completely discontinued therapy and those who were fully adherent. Third, we modeled the discontinued group compared with the full adherence group. For this model, we were interested in assessing the differences between those who had completely discontinued therapy and those who were fully adherent.

Variables examined were based on previously published articles on adherence and those associated with perceived risk and potential exposure to *B. anthracis* spores in this setting. Inhalational anthrax developed in employees who worked on a sorter machine and in the government mail section of the DCPDC (2). Variables included age, sex, race, perceived risk of breathing in *B. anthracis* spores, work location during exposure period, work description during the time of interview, trouble remembering to take pills, experiencing anxiety, physical signs of stress, severity of adverse effects, and adverse effects negatively affecting work performance. For all analysis, SAS 8.2 (SAS Institute, Inc., Cary, NC) was used. For univariate analysis, two-tailed p values were calculated by chi-square test for dichotomous variables. Potential covariates for the logistic regression models included those with p<0.20 in univariate analysis, and possible confounders. We followed a backward elimination strategy to remove nonsignificant covariates in building final parsimonious models. A p<0.05 was determined to be statistically significant.

For all qualitative and quantitative interviews, workers were informed that their participation was voluntary and anonymous. Anthrax infections did not develop in any of the workers who participated in the interventions or interviews.

## Results

### Characteristics of Participants

Of 251 DCPDC workers invited to participate in the questionnaire, 245 (98%) agreed. Among participants, 124 (51%) were male, and 214 (88%) identified themselves as black. Only 1 (0.5%) worker was 18–24 years of age, 74 (30%) were 25–44 years, 163 (67%) were 45–64 years, and 6 (2%) were >65 years of age.

### Comparison of Adherence among Workers

Among those who completed the questionnaire, 98 (40%) reported full adherence, 45 (18%) discontinued prophylaxis and never restarted, and 102 (42%) were classified as intermediate. Overall, 186 (76%) workers were taking prophylaxis at the time of the interview, including 88 (86%) of the 102 classified as in the intermediate group. Among the intermediate group, 14 (14%) reported discontinuing prophylaxis and restarting at least once, but they were not taking antibiotics at the time of the interview. A total of 45 workers from the discontinued group and 48 workers from the intermediate group reported stopping prophylaxis.

Among the 102 workers classified as intermediate, 40 (39%) reported ever reducing the dosage, 65 (64%) forgot to take at least one pill the previous day, and 48 (47%) reported discontinuing prophylaxis and restarting at least once. Among those who restarted, 20 (42%) missed at least one pill the previous day, and 22 (46%) reported they had ever reduced the dosage.

We examined reasons for stopping prophylactic antimicrobial therapy (Table 1). Most workers reported that several factors influenced their decision to discontinue prophylaxis; 60% cited five or more reasons. Trouble managing adverse effects to antimicrobial agents was the most common reason. Concern over possible long-term adverse effects associated with prolonged antimicrobial therapy was the second most common reason for stopping. Similar reasons were given by the workers who reported reducing the dosage of the prescribed antimicrobial therapy. Workers who stopped therapy also reported lacking sufficient information about anthrax and antimicrobial therapy, specifically, information from USPS or CDC.

**Table 1:** Reasons for stopping prophylaxis or reducing dosage during anthrax outbreak, Washington, D.C., 2001

Reasons for stopping prophylaxis (n=93)^a^	n (%)
Adverse effects	73 (78)
Potential long-term adverse effects	59 (63)
Low risk of developing anthrax disease	47 (51)
Concerns about antibiotic resistance	32 (34)
Negative environmental test results (facility or nasal)	28 (30)
Saving antibiotic for later use	25 (27)
Restrictions to diet or alcohol consumption	22 (24)
Lack of support at work	16 (17)
Difficulty getting appointment with health-care provider	9 (10)
Advised by health-care provider	7 (7)
Expense of health-care provider visit or antibiotic	6 (6)
Reasons for reducing dosage (n=53)^b^	
Adverse effects	38 (72)
Potential long-term adverse effects	8 (15)
Advised by health-care provider	2 (4)
Difficulty remembering to take antibiotic	2 (4)
Take only on workdays	2 (4)
Low supply of pills	1 (2)

^a^Workers were asked to respond to each reason. A total of 45 workers from the discontinued group and 48 workers from the intermediate group reported stopping prophylaxis. ^b^Workers chose only answers that applied. A total of 13 workers from the discontinued group and 40 workers from the intermediate group reported reducing the dosage. Among the 53 workers who reduced their dosage, 5 reported more than one reason, and 5 reported other reasons not included here.

### Predictors of Nonadherence

We wanted to understand the differences between those who were not fully adherent, excluding those who completely discontinued therapy, compared with those who were fully adherent. We therefore modeled intermediate adherence compared with full adherence. Characteristics of these populations and univariate analysis are in Table 2. Independent predictors of intermediate adherence included experiencing “a lot” of adverse effects to antimicrobial therapy, trouble remembering to take pills, as well as age <45 years (Table 3). Experiencing “a lot” of adverse effects, trouble remembering to take pills, and age <45 years were also risk factors for nonadherence in a model combining the intermediate adherence and discontinued groups compared with full adherence (data not shown).

**Table 2:** Characteristics of postal workers with intermediate and full adherence to prophylaxis for inhalational anthrax, Washington, D.C., 2001^a^

Characteristics	Intermediate (n=102) n (%)	Full (n=98) n (%)	RR (95% CI)	p value
Sex^b^				
Female	43 (42)	52 (54)	0.8 (0.6, 1.05)	n.s
Male	59 (58)	45 (46)	Ref	-
Age,^b^ (y)				
18–44	34 (33)	16 (16)	2.0 (1.2, 3.4)	p<0.05
>45	68 (67)	81 (84)	Ref	-
Race/ethnicity^b^				
Black	90 (88)	88 (91)	1.0 (0.9, 1.1)	n.s.
Other	5 (5)	3 (3)	1.6 (0.4, 6.5)	n.s.
White	7 (7)	6 (6)	Ref	-
Work description at interview^c^				
Driver	12 (12)	8 (8)	1.4 (0.6, 3.3)	n.s.
Government mail	17 (16)	21 (21)	0.8 (0.4, 1.4)	n.s.
Administration	7 (7)	6 (6)	1.1 (0.4, 3.2)	n.s.
Plant floor	66 (65)	63 (64)	Ref	-
Worked on sorter or in government mail section^d^				
Yes	70 (72)	70 (75)	0.9 (0.8, 1.1)	n.s.
No	27 (28)	23 (25)	Ref	-
Perceived risk^e^				
High	58 (57)	60 (61)	0.9 (0.7, 1.2)	n.s.
Some	39 (38)	35 (36)	1.1 (0.7, 1.5)	n.s.
None	5 (5)	3 (3)	Ref	-
Adverse effects^f^				
A lot	20 (20)	9 (9)	2.1 (1.02, 4.4)	p<0.05
Some	54 (53)	48 (49)	1.1 (0.8, 1.4)	n.s.
Not at all	28 (27)	41 (42)	Ref	-
Physical signs of stress^g^				
5–11 signs	37 (36)	28 (29)	1.3 (0.8, 1.9)	n.s.
1–4 signs	50 (49)	57 (58)	0.8 (0.6, 1.1)	n.s.
0 signs	15 (15)	13 (13)	Ref	-
Anxiety^h^				
Yes	37 (36)	33 (34)	1.1 (0.7, 1.6)	n.s.
No	65 (64)	65 (66)	Ref	-
Trouble remembering pills^i^				
Yes	67 (66)	44 (45)	1.5 (1.1, 1.9)	p<0.05
No	35 (34)	54 (55)	Ref	-
Worse work performance^j^				
Yes	16 (16)	15 (15)	1.0 (0.5, 1.9)	n.s.
No	86 (84)	83 (85)	Ref	-

^a^RR, relative risk; 95% CI, 95% confidence interval; n.s., not statistically significant; ref, referent. ^b^One missing value for full adherence. ^c^Work location during the survey interview, December 18–20, 2001. ^d^Worked close to these areas for more than half of the normal workdays during exposure period of October 12–21, 2001. Responses of “don’t know” were excluded from analysis (n=13). ^e^Perceived risk of breathing in *Bacillus anthracis* spores during exposure period of October 12–21, 2001. ^f^Reported how much side effects affected their lives. ^g^Physical signs of stress included fatigue, headaches, chest pain, rapid heartbeat, unplanned changes in weight, less sleep or difficulty sleeping, muscle tremors or twitches, difficulty or rapidity in breathing, elevated blood pressure, nausea or vomiting, and dizziness or lightheadedness. ^h^Reported they experienced anxiety since anthrax events started. Anxiety was one of 22 listed physical, emotional, mental, and behavioral signs of stress on our questionnaire. ^i^Reported they sometimes or almost always had trouble remembering their pills. ^j^Reported side effects negatively affected their work performance.

**Table 3:** Predictors of intermediate adherence (n=102) compared with full adherence, Washington, D.C., 1991 (n=98)^a^

Predictor covariates		Adjusted, OR (95% CI)	p value
Age	18–44 y	2.2 (1.1, 4.4)	p<0.05
Adverse effects^b^	A lot	2.8 (1.1, 7.4)	p<0.05
	Some	1.5 (0.8, 2.8)	n.s.
	Not at all	ref	-
Trouble remembering pills^c^	Yes	2.2 (1.2, 4.1)	p<0.05


^a^ OR, odds ratio; 95% CI, 95% confidence interval; n.s., not statistically significant; ref, referent. ^b^Reported how much side effects affected their life. ^c^Reported they sometimes or almost always had trouble remembering their pills.

We wanted to understand the differences between those who completely discontinued therapy and those who were fully adherent. We therefore modeled the discontinued group compared with the full adherence group. Characteristics of these populations and univariate analysis can be found in Table 4. Independent predictors of discontinuing therapy included experiencing “a lot” of adverse effects, anxiety, and age <45 years (Table 5). Those workers who reported a high perceived risk of having breathed in *B. anthracis* spores during October 12–21, 2001, were significantly less likely to have discontinued therapy. Those who experienced five or more physical signs of stress were also significantly less likely to have discontinued therapy.

**Table 4:** Characteristics of postal workers who discontinued or were fully adherent to prophylaxis for anthrax, Washington, D.C., 2001^a^

Characteristics	Discontinued (n=45) n (%)	Full adherence (n=98), n (%)	RR (95% CI)	p value
Sex^b^				
Female	25 (56)	52 (54)	1.0 (0.7, 1.4)	n.s.
Male	20 (44)	45 (46)	Ref	-
Age^b^				
18–44 y	25 (56)	16 (16)	3.4 (2.0, 5.7)	p<0.05
>45 y	20 (44)	81 (84)	Ref	-
Race/ethnicity^b^				
Black	36 (80)	88 (91)	0.9 (0.7, 1.04)	n.s.
Other	3 (7)	3 (3)	2.2 (0.4, 10.4)	n.s.
White	6 (13)	6 (6)	Ref	-
Work description at interview^c^				
Driver	6 (13)	8 (8)	1.6 (0.6, 4.4)	n.s.
Government mail	3 (7)	21 (21)	0.3 (0.1, 0.99)	p<0.05
Administration	12 (27)	6 (6)	4.3 (1.7, 10.9)	p<0.05
Plant floor	24 (53)	63 (64)	Ref	-
Worked on sorter or in government mail section^d^				
Yes	18 (43)	70 (75)	0.6 (0.4, 0.8)	p<0.05.
No	24 (57)	23 (25)	Ref	-
Perceived risk^e^				
High	16 (35)	60 (61)	0.6 (0.4, 0.9)	p<0.05
Some	25 (56)	35 (36)	1.5 (1.1, 2.2)	p<0.05
None	4 (9)	3 (3)	Ref	-
Adverse effects^f^				
A lot	11 (25)	9 (9)	2.7 (1.2, 6.0)	n.s.
Some	19 (42)	48 (49)	0.9 (0.6, 1.3)	n.s.
Not at all	15 (33)	41 (42)	Ref	-
Physical signs of stress^g^				
5–11 signs	7 (16)	28 (29)	0.5 (0.2, 1.1)	n.s.
1–4 signs	29 (64)	57 (58)	1.1 (0.8, 1.4)	n.s.
0 signs	9 (20)	13 (13)	Ref	-
Anxiety^h^				
Yes	17 (38)	33 (34)	1.1 (0.7, 1.8)	n.s.
No	28 (62)	65 (66)	Ref	-
Trouble remembering pills^i^				
Yes	23 (51)	44 (45)	1.1 (0.8, 1.6)	n.s.
No	22 (49)	54 (55)	Ref	-
Worse work performance^j^				
Yes	9 (20)	15 (15)	1.3 (0.6, 2.7)	n.s.
No	36 (80)	83 (85)	Ref	-

^a^RR, relative risk; 95% CI, 95% confidence interval; n.s., not statistically significant. ^b^One missing value for full adherence. ^c^Work location during the survey interview, December 18–20, 2001. ^d^Worked close to these areas for more than half of the normal workdays during exposure period of October 12–21, 2001. Responses of “don’t know” excluded from analysis (n=13). ^e^Perceived risk of breathing in *Bacillus anthracis* spores during exposure period of October 12–21, 2001. ^f^Reported how much side effects affected their lives. ^g^Physical signs of stress included fatigue, headaches, chest pain, rapid heartbeat, unplanned changes in weight, less sleep or difficulty in sleeping, muscle tremors or twitches, difficulty or rapidity in breathing, elevated blood pressure, nausea or vomiting, and dizziness or lightheadedness. ^h^Reported they experienced anxiety since anthrax events started. Anxiety was one of 22 listed physical, emotional, mental, and behavioral signs of stress on our questionnaire. ^i^Reported they sometimes or almost always had trouble remembering their pills. ^j^Reported side effects negatively affected their work performance.

**Table 5:** Predictors of discontinued Therapy (n=45) compared with full adherence (n=98), Washington, D.C., 2001^a^

Predictor covariates		Adjusted, OR (95% CI)	p value
Age	18-44 y	6.7 (2.6 to 17.3)	<0.05
Perceived risk^b^	High	0.1 (0.01 to 0.8)	<0.05
	Some	0.4 (0.1 to 3.0)	n.s.
	None	ref	-
Adverse effects^c^	A lot	20.4 (3.0 to 140.1)	<0.05
	Some	1.7 (0.6 to 5.1)	n.s.
	Not at all	ref	-
Physical signs of stress^d^	5-11 signs	0.02 (0.003 to 0.2)	<0.05
	1-4 signs	0.3 (0.1 to 1.1)	n.s.
	0 signs	Ref	-
Anxiety^e^	Yes	3.5 (1.1 to 10.9)	<0.05

^a^OR, odds ratio; 95% CI, 95% confidence interval; n.s., not statistically significant; ref, referent. ^b^Perceived risk of breathing in *B. anthracis* spores during exposure period October 12-21, 2001. ^c^Reported how much side effects affected their life. ^d^Physical signs of stress included fatigue, headaches, chest pain, rapid heartbeat, unplanned changes in weight, less or difficulty sleeping, muscle tremors or twitches, difficulty or rapid breathing, elevated blood pressure, nausea or vomiting, and dizziness or lightheadedness. ^e^Reported they experienced anxiety since anthrax events started.  Anxiety was one of 22 listed symptoms of stress on our questionnaire.

### Postal Workers’ Experiences and Qualitative Evaluation of Interventions

A total of 38 workers participated in five focus groups, and 22 participated in individual qualitative interviews. The age, sex, and race/ethnic characteristics of qualitative interview participants were similar to those of respondents to the survey questionnaire.

When asked in focus groups and individual qualitative interviews about what adherence interventions were helpful, workers consistently cited repeated visits by public health staff to worksites. Workers reported that the ability to ask personal questions and the distribution of various materials covering multiple health- and work-related issues helped workers complete prophylaxis and promoted adherence by providing accurate and needed information about anthrax, antimicrobial therapy, risk for disease, and the outbreak investigation. Workers reported that this information helped reduce their stress levels and motivated them to continue prophylaxis.

Workers recalled receiving little information at the free antimicrobial distribution sites, and some had forgotten or misunderstood the initial information given. Several opportunities to speak with public health staff were necessary to clarify questions, especially as new issues arose. However, some workers complained that public health staff could not provide adequate answers to all their questions, such as those related to the long-term status of viable *B. anthracis* spores inhaled into the lung, the long-term effects of extended antimicrobial therapy, environmental sampling results, the need for personal protective gear, and other occupational health concerns.

In the questionnaire, 82% of workers reported they wanted to receive public health information in a variety of formats, including both orally and written, as well as information from the media. The questionnaire showed that only 3% of workers did not participate in oral communication interventions, 2% did not receive written materials distributed to employees at the worksite or at their homes, and 21% did not see posted signs and messages at work.

## Discussion

After the first bioterrorism-related anthrax outbreak in the United States, we rapidly developed and implemented multiple adherence interventions to prevent inhalational anthrax in >2,000 DCPDC workers. This was the first time adherence interventions have been conducted and evaluated in an applied public health bioterrorism response. Our interventions promoted the message that adherence was essential for the full 60 days of antimicrobial therapy. Further, the interventions were carried out during the entire 60-day period. Seventy-six percent of postal workers were taking antimicrobial prophylaxis at the time of the evaluation. Despite differences in assessing adherence, the adherence found in this study was relatively high compared with other studies of adherence to short-course antimicrobial therapy. For example, Ley (15) reported approximately 50% adherence in a review of adherence studies to short-course antibiotics, and Brookoff (16) reported only 31% adherence to a 10-day course of doxycycline (n=386) for outpatient treatment of pelvic inflammatory disease.

Many issues hindered adherence in this anthrax outbreak, including adverse effects of the antimicrobial prophylaxis, such as gastrointestinal upset and yeast infection, trouble remembering to take the pills, perceived risk, anxiety, and physical signs of stress. Although these factors occurred in the context of a bioterrorism event, similar adherence obstacles have been reported elsewhere (5,7,17,18). Additional issues complicating adherence among postal workers included the large number of workers affected, occupational health and other work-related issues, limited capacity of local departments of health to undertake a program to promote adherence for a large number of people in an emergency, and the hysteria and media coverage associated with this bioterrorism event, which likely magnified miscommunication and workers’ confusion.

In developing the intervention protocols, we drew upon lessons learned from adherence strategies for isoniazid treatment for latent tuberculosis infection and highly active antiretroviral therapy for HIV infection. Studies of these strategies conclude that interventions must be multifaceted, ongoing, flexible, individualized, and repetitive to achieve optimal adherence levels (5,8,9,18–20). Our interventions included many of these characteristics, such as repeated visits, clarifying questions, counseling workers, incorporating pill-taking into daily routines, and providing workers with as much information as possible about anthrax and antimicrobial therapy. Inhalational anthrax as a disease and bioterrorism-associated disease are complex issues and relaying this information to people was difficult. Therefore, multiple formats (verbal, written, and graphic) were necessary to effectively communicate information to workers.

Many workers mistook signs of stress (e.g., complaints of fatigue, lack of sexual drive, and increased crying) for adverse effects of the antimicrobial therapy. Further, the stress associated with the bioterrorist event magnified the adverse effects associated with prophylaxis. For some symptoms, distinguishing between adverse effects of stress and those of the antimicrobial therapy, such as gastrointestinal upset, was impossible. Those who worked close to areas where coworkers with inhalational anthrax had worked reported more physical signs of stress, had a higher perceived risk of having breathed in *B. anthracis* spores, and were also more likely to have continued therapy. Those who had anxiety were more likely to have discontinued therapy. Published articles report associations between anxiety or depression and nonadherence (7,17), and some researchers posit that the inability to cope with anxiety is the better predictor of nonadherence (17). These findings highlight the importance of communicating early and repeatedly the known adverse effects people should expect, and how to manage all potential effects, including those caused by prophylaxis and stress or anxiety related to bioterrorist events.

Only self-reports were collected to assess adherence in this evaluation. Several studies suggest that self-reporting overestimates adherence, while reports of nonadherence are usually valid (5,7). Therefore, our results may have overestimated adherence, but it is unlikely that we overestimated the number of persons who discontinued prophylaxis. Data were collected from a convenience sample and may not be representative of all DCPDC workers. A March 2002 phone survey among DCPDC workers (62% response rate) reported similar age, sex, and race/ethnicity characteristics (21). Because we did not have a control group who did not receive interventions to promote adherence, we cannot measure the effectiveness of our interventions; however, our adherence findings were similar to those of other studies that were not implemented in the setting of a bioterrorist emergency response (7,8,11). In addition, the evaluation was conducted during the holiday season, the busiest time of the year for the USPS, and we were permitted to conduct the questionnaire only with workers on the day shift (7 a.m.–3 p.m.). The experiences of day-shift workers may be different from those who work other shifts, although, based on the qualitative findings carried out with workers from all shifts and the continual interactions with workers throughout the 60-day period, these findings likely reflect the experiences of most DCPDC workers. Last, our evaluation may have been affected by the general media coverage of the bioterrorism events.

Nonadherence is common and should be expected in all settings, especially in a bioterrorism-related context that involves further challenges and complications to adherence. Considering the large number of workers who took less than the recommended regimen, evaluating adherence promotion interventions during bioterrorist outbreaks is very important. In emergency settings, adherence programs may overburden local departments of health because they require ongoing personal interactions and are labor-intensive when large numbers of people are affected. Efforts to develop a plan to promote adherence in the event of a bioterrorism outbreak, which could be tailored to the situation and implemented immediately, will aid future public health emergency responses where adherence to recommended prophylaxis is necessary to save lives. During occupational exposures, supplementing occupational health resources may be necessary. To optimally promote adherence, such plans should incorporate continual interaction with the affected persons, provide consistent and clear messages, and include interventions that help persons incorporate pill-taking into daily routines and manage known adverse effects, including those caused by prophylaxis, anxiety, and stress related to bioterrorism events.
